# Commentary: Predicting histologic grades for pancreatic neuroendocrine tumors by radiologic image-based artificial intelligence: a systematic review and meta-analysis

**DOI:** 10.3389/fonc.2025.1456557

**Published:** 2025-03-31

**Authors:** Elettra Merola, Andrea Michielan, Armando Gabbrielli

**Affiliations:** ^1^ Gastroenterology Unit, Giovan Battista (G.B.) Grassi Hospital (Azienda Sanitaria Locale (ASL) Roma 3), Rome, Italy; ^2^ Azienda Provinciale per i Servizi Sanitari (APSS), Gastroenterology and Digestive Endoscopy Unit, Santa Chiara Hospital, Trento, Italy; ^3^ Center for Medical Sciences (CISMed), University of Trento, Trento, Italy

**Keywords:** artificial intelligence, radiomics, pancreatic neuroendocrine tumors, grading, meta analysis

## 1 Introduction

Artificial intelligence (AI) is currently in the spotlight, and its use in the medical field is exponentially increasing. Its application with radiomics enables different techniques, such as “deep learning” and “machine learning,” to translate radiological features into histological information (1). This innovation may significantly affect patient management by reducing the number of required tests to achieve a diagnosis and avoiding invasive and risky procedures to obtain tissue samples.

The field of neuroendocrine neoplasms (NENs) is progressively incorporating AI applications. These neoplasms are very heterogeneous, with the primary tumor site potentially involving all human organs, and with a prognosis that depends on several factors, including Ki67/grading, stage, and differentiation (2). Although initially considered a very rare entity, their incidence and prevalence have increased over time, primarily due to advancements in technology (3). For NENs, AI may represent a further step towards personalized patient management. However, there is a dearth of high-quality studies on this topic in the literature.

## 2 Comment on the findings and discussion

Yan et al. (4) investigated the ability of radiological image-based AI models to predict the grading of pancreatic neuroendocrine tumors (PanNETs) through a meta-analysis of 20 retrospective studies (2,639 patients) (4). They reported a pooled positive likelihood ratio of 4.382, and a pooled negative likelihood ratio of 0.215. Based on these encouraging results, the authors highlighted the potential value of AI in spite of standard histology. This is because the proliferation index (and thus grading) may be expressed within each lesion with heterogeneity, and a biopsy from a spot of one lesion may fail to represent the entire neuroendocrine disease affecting a patient.

Although we agree that AI may make a significant contribution to the diagnostic algorithm of PanNETs, we also believe that current expectations from AI in this field are too high. As correctly pointed out by Yan et al. (4), a high heterogeneity was observed in the studies of the meta-analysis (I^2^ = 90.42%, P<0.05) due to several factors, including a lack of external validation, the adoption of different imaging modalities, and different modeling algorithms (4). The fact that NEN management is often based on expert opinions in a real-world setting justifies some of these limitations. For example, although guidelines clearly indicate which tests to perform for diagnosis (5, 6), standardized protocols regarding follow-up strategies are still lacking (7). This consideration may explain why some papers in the meta-analysis utilized CT while others employed MRI for image-based AI (4).

Furthermore, AI’s ability to predict PanNET grading still seems to be imprecise. Some studies have shown that AI only differentiated between G1 and G2/G3 cases, and in others only between G1/G2 and G3 (4). These results are very limited since a different grading corresponds to a different therapeutical approach. In details, G2 tumors with Ki67 of 15%-20% are expected to behave more aggressively than Ki67 < 3%, and this might for example suggest to adopt chemotherapy or target therapy instead of somatostatin analogs. Moreover, the WHO 2017 classification has further divided the G3 category into two subgroups based on tumor differentiation: neuroendocrine carcinomas (NECs) and NETs G3 (8). These subgroups correspond to two different diseases, with different therapies to be administered and different prognoses. In detail, for metastatic cases, NECs are treated at first-line with platinum-etoposide chemotherapy, while NETs G3 generally benefit from other treatments (i.e., temozolomide/capecitabine, FOLFOX, etc.). While the median overall survival for NECs is typically 11-12 months, studies report a median rate of 31.9 months for NETs G3 (9). These data clarify why the distinction between NECs and NETs G3 cannot be ignored.

For these reasons, our opinion is that AI has yet to establish a role in defining NEN grading. This application may serve as a “salvage” strategy in very select cases when a procedure to achieve histological diagnosis is risky due to serious comorbidities.

In a commentary regarding the role of AI in the patient management, potential risks associated with overreliance on AI for clinical decision-making cannot be ignored. As mentioned above, a misclassification of NENs (both in terms of grading/ki67 and differentiation) might lead to a wrong therapeutical approach and thus to a worse prognosis. The use of AI in the clinical setting is also affected by significant ethical concerns regarding privacy and data security, transparency, clinical validation, and professional responsibility (10, 11). Potential biases need to be considered as well (i.e., data bias, environmental bias, algorithmic bias), since they may prevent study replicability and influence the reported outcomes. In particular, algorithmic bias may affect equity in healthcare delivery, misrepresenting and discriminating specific subgroups of patients with the risk of disparities in their diagnosis, treatment, and outcomes. Regarding this ethical issue, the use of diverse datasets including data from underrepresented groups is helpful in considering the diversity present in the real-world setting. Furthermore, human interpretation and intervention are also needed to mitigate all these limitations (11).

In conclusions, we agree that AI may represent a unique support in the medical and scientific field, but we also think that published data are still scanty and affected by significant biases, and that only high-quality, prospective studies will allow researchers to draw any definitive conclusions regarding its clinical applications.
